# Socioeconomic and demographic determinants of tobacco use in Kenya: A secondary data analysis of findings from the Kenya Demographic and Health Survey 2022

**DOI:** 10.18332/tid/210324

**Published:** 2025-11-04

**Authors:** Peter O. Magati, Jeffrey Drope, Raphael Lencucha, Starley B. Shade, Jerry John Ouner, Francesca Odhiambo, Stella Bialous

**Affiliations:** 1 Development Hub, Health Policy Division, Nairobi, Kenya; 2 Economics for Health, Johns Hopkins Bloomberg School of Public Health, Baltimore, United States; 3 Faculty of Medicine, School of Physical and Occupational Therapy, McGill University, Montreal, Canada; 4 Institute for Global Health Sciences, Department of Epidemiology and Biostatistics, University of California, San Francisco, United States; 5 Department of Family Health Care Nursing, School of Nursing, University of California, San Francisco, United States; 6 Kenya Medical Research Institute, Centre for Microbiology Research, Kisumu, Kenya; 7 Department of Social and Behavioral Sciences, School of Nursing, University of California, San Francisco, United States

**Keywords:** tobacco use, prevalence, tobacco control, older adults

## Abstract

**INTRODUCTION:**

Tobacco use is a major public health crisis in Kenya, leading to over 6000 deaths annually. With a significant number of young people and adults using tobacco, the nation faces a rising health burden. The Kenyan government has implemented educational programs to curb consumption. This study analyzes data from the 2022 Kenya Demographic and Health Survey (KDHS) to assess changes in tobacco use from 2014 to 2022 and identify key demographic and socioeconomic determinants.

**METHODS:**

This study is a secondary data analysis of the 2022 Kenya Demographic and Health Survey (KDHS), a nationally representative survey of 46609 adults (aged 15–54 years). Data access was through the MEASURE DHS platform, ensuring ethical handling. A logistic regression model was used to estimate odds ratios of tobacco use, adjusting for socioeconomic and demographic factors. The analysis accounted for the survey's complex design using survey weights and clustering and was conducted in Stata 17 software.

**RESULTS:**

Between 2014 and 2022, overall tobacco use declined. Among men, prevalence dropped from 17.3% to 12.81% (25.95% decrease), and among women from 3.10% to 2.64% (14.84% decrease). While women's smoking slightly increased (0.18–0.35%), their smokeless use decreased (0.93–0.77%). Tobacco use was linked to age, marital status, residence, region, education level, and gender. Men's tobacco use odds increased with age, with those aged 20–24 years nearly five times more likely to use tobacco than those aged 15–19 years (AOR=4.44; 95% CI: 4.44–4.44). Married men were less likely to use tobacco than divorced, separated, or widowed men.

**CONCLUSIONS:**

The observed declines in tobacco use, especially among males, suggest that current tobacco control efforts are positively impacting public health. Given the financial strain of health costs, preventive interventions are crucial. Research on socioeconomic and demographic factors can guide targeted behavioral change strategies. Continued policy measures like increased tobacco taxation, raising the legal sale age, and enforcing advertising bans and smoke-free policies remain essential to further reduce tobacco's health burden in Kenya.

## INTRODUCTION

Tobacco use is one of the leading causes of death globally, accounting directly for nearly 8 million deaths – 80% of which are in low-to-middle income countries (LMICs)^1,2^. It is expected that by 2030, tobacco use will produce the highest burden of premature mortality and disability in the world compared to other health risk factors with LMICs being more affected by this burden than high income countries^3^. In Kenya, every year, more than 6000 people die of tobacco related diseases, while more than 220000 children and more than 2737000 adults continue to use tobacco each day^4^. Kenya, like other countries in Sub-Saharan Africa, has a relatively low prevalence rate of tobacco use compared to countries in other regions. However, with a youthful population that is growing rapidly, a decline in prevalence may not translate to a decline in the number of tobacco users, even though the country is expected to experience a decrease in prevalence^5,6^. In addition to the youthful population, the Kenyan population is experiencing a rise in average household income. Together with the increased presence of tobacco industry and its products, there is a risk of greater tobacco use. This suggests a heavy burden of tobacco-related diseases and deaths in the future, unless tobacco control measures are strengthened.

Kenya remains an active supporter of the WHO Framework Convention on Tobacco Control (FCTC) and since ratifying the treaty in 2004 passed the Tobacco Control Act (TCA) in 2007 to control the production, manufacture, sale, labeling, advertising, promotion and sponsorship of tobacco products. Despite active industry opposition and litigation, the regulations were finalized in 2016. These regulations include interventions such as a ban on cigarette advertisement, sale of cigarettes to minors, sale of single stick cigarettes to consumers, and tobacco use (in particular smoking) in public areas; as well as the creation of a solatium fund for tobacco control research, rehabilitation and cessation programs^7-9^.

In Kenya, while plans have been developed to combat tobacco use – e.g. the Tobacco Control Action Plan 2010–2015, the Strategy for Prevention and Control of Non-Communicable Diseases (NCDs) 2015–2020 and the Kenya National NCD Strategic Plan 2021/22–2025/26 – more analysis is needed to determine the complexities of tobacco consumption among different discrete or semi-discrete population groups. The paucity of data makes it not only challenging to accurately describe consumption trends but also to assess the socioeconomic characteristics of people who consume tobacco products and assess the progress of tobacco control measures to date. Understanding these determinants and factors is important to develop intervention policies because scarce resources will be then more prudently directed towards the socioeconomic groups with higher needs.

The objective of this research is to provide an update of earlier research that used the 2014 Kenya Demographic and Health Survey (KDHS)^10^ to determine the socioeconomic and demographic determinants of tobacco use in Kenya by using the most recent 2022 results from the same survey. This research also provides an opportunity to identify changes in socioeconomic and demographic variables associated with tobacco use between the two KDHS time periods. The 2014 paper found that for both men and women, tobacco use is influenced by factors such as age, marital status, place of residence, region, education level, and gender. The prevalence of smoking and smokeless tobacco use was found to be higher among men (17.3% and 3.1%, respectively) than women (0.18% and 0.93%, respectively). The paper concludes that resources for tobacco control initiatives should be allocated based on these socioeconomic, demographic, and geographical disparities. This study hence analyzes data from the 2022 Kenya Demographic and Health Survey (KDHS) to assess changes in tobacco use from 2014 to 2022 and identify key demographic and socioeconomic determinants.

## METHODS

### Kenya demographic and health survey

This is a secondary analysis of the 2022 Kenya Demographic and Health Survey (KDHS) data, which builds on previous findings from the 2014 KDHS data^10^. The 2022 KDHS is a nationally representative household survey that interviewed 37911 households (14330 in urban areas and 23581 in rural areas). This study includes a total of 14453 men and 32156 women aged 15–54 years that were asked about tobacco use in the KDHS.

### Data analysis

To account for the complex survey design of the KDHS, which involves stratification, clustering, and unequal sampling probabilities, survey weights and clustering were incorporated into the analysis. This research adopts a logistic regression model due to the binary nature of the key dependent variable, which indicates whether an individual uses tobacco^11,12^. The model estimates the probability of tobacco use based on a set of predictive variables according to:


Pr(y=1|x)=xβ+ε 1

where the dependent variable y is a binary indicator of an individual’s tobacco use status, 1 if the individual reports using tobacco and 0 if not. The set of independent variables *x* includes: 1) age groups in 5-year intervals starting at 15 years, the widely accepted benchmark for ‘adult’ in the tobacco control literature [i.e. 15–19 (ref.), 20–24, … 50–54] and 54 years being the ceiling age of respondents; 2) place of residence [rural or urban (ref.)]; 3) gender (men and women, as defined by the KDHS); 4) marital status [never married (ref.), living together, married, widowed/divorced/separated)]; 5) region with Rift Valley as reference; 6) highest level of education [no education/preschool, primary, secondary or higher education (ref.)]; 7) occupation [unemployed (ref.), agriculture, service, casual laborer]; 8) a dummy for head of household; 9) wealth based on 5 wealth quintiles calculated by the DHS; and 10) self-reported tobacco use (as captured in the DHS questionnaire), including smoking and smokeless tobacco use, and whether the person was staying at his/her permanent home at the time of the interview.

The survey weights were incorporated into the logistic regression model to ensure that the estimated odds ratios reflect the effects of the predictor variables on the level of the overall population, accounting for the unequal selection probabilities of individuals in the sample. Exponentiated coefficients from the model provided odds ratios, indicating how much more (or less) likely individuals with certain characteristics are to use tobacco compared to a reference group, after adjusting for other variables in the model; 95% confidence intervals for the odds ratios were also calculated. Analyses were conducted in Stata (Version 17.0, StataCorp, College Station, TX), utilizing the svyset command to specify the survey design and the svy: logit command to fit the weighted logistic regression model.

The model focuses on socioeconomic determinants and does not include price as part of the independent variables. This is because the KDHS does not collect data on price.

## RESULTS

Overall, tobacco use prevalence in Kenya decreased from 17.3% and 3.10% among men and women in 2014 to 12.81% and 2.64%, respectively, in 2022, a decrease of 25.95% and 14.84% for men and women, respectively.

### Descriptive statistics

The 2022 KDHS study included 62.20% of respondents who resided in rural areas while 37.80% resided in urban areas. The unemployment rate among the male and female respondents was 30.50% and 62.29%, respectively. Education level was similar by gender: 35.9% of males and 36.3% of females indicated that their education level was primary school; 39.5% of men and 38% of female respondents had secondary schooling; and 20.5% of men and 19.1% of female respondents had higher than secondary education.

### Tobacco use prevalence

Among respondents, 12.18% and 2.64% indicated using smoking and smokeless tobacco, respectively, with 8.99% and 0.81% of men and women using tobacco daily (Table 1). The prevalence is even lower among male respondents aged 15–19 years (1.28% smoking and 0.45% smokeless). However, the prevalence increases as the age bracket changes peaking with those at the age of 45–49 years having the highest prevalence at 26.15%. Similarly, smokeless tobacco use increases with age peaking at 4.48% with those aged 35–39 years. Smoking prevalence is similar for urban and rural areas though slightly higher in urban areas. Smokeless tobacco use among males is similar between the two but with rural areas being slightly higher.

**Table 1:** Male and female use of smoking and smokeless tobacco for the general population in Kenya

*Characteristics*	*Men*			*Women*		
	*n*	*Smoking prevalence %*	*Smokeless prevalence %*	*n*	*Smoking prevalence %*	*Smokeless prevalence %*
**Total**	12693	12.18	2.64	32050	0.35	0.77
**Age** (years)						
15–19	3349	1.28	0.45	6404	0.11	0.3
20–24	2332	6.3	1.76	5762	0.28	0.45
25–29	2109	10.57	3.03	5443	0.28	0.75
30–34	1748	15.16	4.12	4561	0.46	1.10
35–39	1628	19.1	4.48	4354	0.25	1.03
40–44	1386	21.57	3.25	3100	0.52	1.03
45–49	1117	23.9	4.21	2532	0.79	1.34
50–54	784	26.15	3.19	6404	0.11	0.3
**Residence**						
Urban	5232	11.10	2.06	12386	0.35	0.36
Rural	9221	12.79	2.97	8188	0.32	1.02
**Marital status**						
Never married	6508	5.58	1.35	10048	0.25	0.23
Living with partner	417	13.43	1.44	1858	0.38	1.45
Married	6650	14.6	3.55	16454	0.3	0.83
Widowed	878	42.14	5.92	3796	0.63	1.58
Divorced	6508	5.58	1.35	10048	0.25	0.23
No longer living together	417	13.43	1.44	1858	0.38	1.45
**Education level**						
No education	837	10.75	17.2	3836	0.94	4.69
Primary	5499	18.26	2.91	11807	0.34	0.41
Secondary	5635	9.14	1.03	11634	0.11	0.09
Higher education	2482	6.08	0.81	4879	0.35	0.18
**Occupation**						
Unemployed	2921	2.81	1.99	14718	0.35	0.88
Agriculture	218	8.72	4.13	159	0	0
Service/manual	4455	16.05	2.22	3994	0.35	1.03
Non-manual	1982	7.42	1.11	4758	0.32	0.34
**Region**						
Coast	1851	16.05	2.11	4001	0.52	0.6
Northeastern	920	4.46	3.7	2109	0.24	0.14
Eastern	2415	23.48	3.89	4969	0.46	0.72
Central	1408	19.74	2.41	2949	0.37	0.17
Rift Valley	4312	8.49	3.62	9777	0.31	1.73
Western	1341	7.9	0.6	3140	0.19	0.1
Nyanza	1832	3.93	0.55	4267	0.09	0.09
Nairobi	374	8.82	1.87	944	0.64	0.32
**Wealth quantiles**						
Poorest	3030	14.98	7.16	7073	0.52	2.83
Poorer	2828	15.03	1.84	5742	0.24	0.24
Middle	3045	12.22	1.51	6345	0.25	0.22
Richer	3309	10.97	1.57	7160	0.27	0.14
Richest	2241	6.51	0.67	5836	0.34	0.15

Men who are never married or divorced have the lowest tobacco use prevalence, compared to other marital status categories.

Smokeless tobacco use among men also varies with education level. Those with no education have a higher consumption of smokeless tobacco at 17.20% and this decreases as the education level increases with post-secondary educated men having a smokeless prevalence of 0.81%. Smoking prevalence is 10.75% for those with no formal education and increases to 18.26% for those with primary schooling. Prevalence drops as the education level increases thereafter, dropping to 6.08% for those with a higher education qualification.

Male smoking prevalence is highest among those who are employed in the service-manual sectors at 16.05% while those in non-manual sector have a prevalence rate of 7.42%. Male respondents that are unemployed have the lowest prevalence rate of 2.81%. A similar trend is seen for smokeless tobacco with the unemployed having a prevalence of 1.99%.

Counties in the Eastern region have the highest prevalence rates for male smokers at 23.48%, followed by the Central counties where the smoking prevalence is 19.74%. Smokeless tobacco among men is high in counties in Eastern, Northeastern and Rift Valley regions having a prevalence of 3.89%, 3.70% and 3.62%, respectively.

The data indicate a negative correlation between wealth and tobacco use, with the poorest individuals exhibiting the highest prevalence of both smoking and smokeless tobacco use.

Among the female KDHS respondents, smoking prevalence is 0.35% while the smokeless tobacco prevalence is 0.77% (Table 1). Although the prevalence was low compared to men, some similar patterns by demographic characteristics were observed. For instance, for smokeless, the consumption tends to increase as the respondents get older. However, the prevalence of smoking increases from 0.11% at 15–19 years, with no clear pattern and peaks at 0.79% among those aged 45–49 years. Female respondents with no education have the highest prevalence of smokeless tobacco at 4.69% with the prevalence of smokeless tobacco use decreasing as the education level increases. A similar pattern is observed for female smoking, with the highest rates (0.94%) among those with no formal education.

Results also suggest that while tobacco use among women is still low, prevalence varies across regions. Regionally, Nairobi County has the highest smoking prevalence among women at 0.64% followed by the coastal region at 0.52%. Smokeless tobacco use is highest among women in the Rift Valley with a prevalence rate of 1.73%. This is followed by Eastern at 0.72% and Western at 0.32%. Other regions have lower smokeless prevalence among women.

### Determinants of tobacco use

The multivariable analysis of smoking and smokeless tobacco use, stratified by gender, reveals interesting and statistically significant findings. Table 2 displays the adjusted odds ratios (AORs) and 95% confidence intervals (CIs) for the full sample. The analysis shows that age is a significant determinant of tobacco use for both men and women, with the odds of smoking generally increasing with age. For instance, a man aged 45–49 years has a 4.18 adjusted odds ratio for smoking compared to a man aged 15–19 years, which is a highly significant association (p<0.01). Marital status also plays a role, as married women have a 0.36 adjusted odds ratio for smoking compared to never-married women (p<0.05), suggesting that being married is a protective factor against smoking. The impact of education on smoking odds is more pronounced for men, while its impact on smokeless tobacco use is more significant for women. The findings also highlight significant regional disparities and the influence of socioeconomic factors on tobacco use.

**Table 2:** Adjusted odds ratios and 95% confidence intervals of smoking and smokeless tobacco use, male and female DHS respondents

*Variables*	*Male smoking*		*Male smokeless*		*Female smoking*		*Female smokeless*	
	*AOR*	*95% CI*	*AOR*	*95% CI*	*AOR*	*95% CI*	*AOR*	*95% CI*
**Age** (years)								
15–19 ®	1		1		1		1	
20–24	0.68***	0.64–0.79	4.53***	2.51–8.16	3.63***	1.45–9.11	1.12	0.59–1.75
25–29	1.39***	1.23–1.57	6.49***	3.60–11.69	4.01***	1.49–11.24	1.3	0.76–1.86
30–34	3.19***	3.16–3.20	6.49***	3.53–12.06	6.17***	2.34–16.32	1.43	0.83–2.22
35–39	3.63***	3.63–3.63	6.89***	3.74–12.78	3.35**	1.16–9.77	1.25	0.66–1.98
40–44	3.86***	3.86–3.86	5.16***	2.69–9.77	6.96***	2.53–19.10	1.42	0.73–2.11
45–49	4.18***	4.18–4.18	6.11***	3.22–11.73	10.49***	3.90–28.22	1.65	0.96–2.17
50–54	4.44***	4.44–4.44	5.21***	2.61–10.33				
**Residence**								
Urban ®	1		1		1		1	
Rural	1.003***	1.003–1.003	1.12	0.89–1.39	1.02	0.66–1.55	1.45	1.03–2.03
**Marital status**								
Never married ®	1		1		1		1	
Living together	0.52***	0.67-1.52	0.51	0.44–1.18	0.66	0.44–1.60	2.12**	1.11–3.97
Married	0.4***	0.41–0.45	0.89	0.66–1.21	0.36**	0.19–0.54	0.97	0.56–1.78
Widowed/divorced/separated	1.48***	0.37–0.41	1.72**	1.17–2.48	0.72	0.34–1.02	1.99**	1.09–3.60
**Education level**								
Secondary or higher ®	1		1		1		1	
No education	0.86	0.71–1.04	12.93	9.58–17.64	3.86***	2.03–8.16	36.21***	21.49–60.79
Primary	1.36***	1.36–1.36	2.97	2.25–3.93	1.86**	1.11–3.12	3.13***	1.79–5.37
**Region**								
Coast	1.54***	1.54–1.54	0.5***	0.35–0.69	1.57	0.98–2.74	0.3***	0.19–0.45
North Eastern	0.55***	0.44–0.69	0.8	0.61–0.95	1.82*	1.13–3.45	0.17***	0.10–0.28
Eastern	1.77***	1.77–1.77	0.68**	0.50–0.93	0.47*	0.39–0.74	0.33***	0.21–0.52
Central	1.49***	1.54–1.54	0.78	0.54–1.11	1.34	0.91–1.95	0.3***	0.15–0.49
Rift Valley ®	1		1		1		1	
Western	0.56***	0.47–0.67	0.22***	0.17–0.44	0.76	0.55–1.48	0.14***	0.07–0.27
Nyanza	0.29***	0.29–0.36	0.21***	0.14–0.39	0.37*	0.24–0.98	0.14***	0.07–0.27
Nairobi	0.68**	0.55–0.94	0.84	0.39–1.80	2.41*	1.10–4.54	0.76	0.32–1.58
**Total,** n	14453		14453		32156		15136	

AOR: adjusted odds ratio. Source: KDHS 2022. Significance level:

*** p<0.001.

** p<0.01.

* p<0.05.

® Reference categories.

To examine more established patterns, the multivariable analysis was focused on the adult population (aged ≥23 years) and is presented in Table 3. The inclusion of occupational status in this model revealed that men in non-manual occupations have significantly lower odds of smoking compared to those in agriculture (AOR=0.42, p<0.01). This means they are about 58% less likely to smoke.

**Table 3:** Adjusted odds ratios and 95% confidence intervals of smoking and smokeless tobacco use, adult DHS respondents

*Variables*	*Male smoking*		*Male smokeless*		*Women smoking*		*Women smokeless*	
	*AOR*	*95% CI*	*AOR*	*95% CI*	*AOR*	*95% CI*	*AOR*	*95% CI*
**Age** (years)								
23–29 ®	1		1		1		1	
30–40	1.38***	n/a	1.13	0.75–1.68	1.04	0.54–2.01	1.06	0.72–1.57
40–50	1.7***	1.70–1.70	1.21	0.79–1.86	2.16*	1.13–4.10	1.36	0.91–2.07
50–59	1.84***	1.84–1.84	0.7	0.35–1.45				
**Marital status**								
Never married ®	1		1		1		1	
Living together	0.5***	0.38–0.65	0.52	0.18–1.50	0.61	0.19–1.80	1.6	0.66–3.86
Married	0.39***	0.35–0.42	0.84	0.55–1.28	0.25***	0.11–0.54	0.6	0.42–0.89
Widowed/divorced/separated	1.49***	n/a	1.54	0.88–2.70	0.71	0.31–1.63	1.52	0.76–3.03
**Region**								
Coast	1.39***	1.39–1.39	0.48***	0.28–0.82	1.61	0.80–3.29	0.28***	0.17–0.45
North Eastern	1.2***	n/a	0.85	0.58–1.25	1.97*	1.20–3.22	0.19***	0.11–0.34
Eastern	1.54***	1.54 1.54	0.44***	0.29–0.65	0.42	0.20–0.89	0.43***	0.26–0.71
Central	1.4***	1.40–1.40	1.31	0.78–2.19	2.16*	1.00–4.67	0.24**	0.12–0.48
Rift valley ®	1		1		1		1	
Western	0.57***	0.50–0.65	0.18***	0.09–0.33	0.79	0.45–1.38	0.09***	0.04–0.19
Nyanza	0.29***	0.2–0.37	0.31***	0.18–0.51	0.2	0.09–0.47	0.07***	0.03–0.19
Nairobi	0.68***	0.56–0.89	0.69	0.33–1.42	1.86	1.05–3.32	0.42***	0.17–1.04
**Residence**								
Urban ®	1		1		1			
Rural	1.06***	1.06–1.06	1.07	0.79–1.45	1.45	0.84–2.50	1.63**	1.06–2.50
**Education level**								
Secondary or higher ®	1		1		1			
No education	0.65***	0.50–0.85	8.85***	6.43–12.18	5.26***	2.59–10.66	42.09***	25.79–68.66
Primary	1.25***	1.25–1.25	2.44***	1.61–3.69	2.36	1.12–4.96	4.48***	2.5–7.84
**Occupation**								
Unemployed ®	1		1		1		1	
Agriculture	0.68**	0.47–0.98	0.66	0.33–1.33	1	n/a	1	n/a
Non-manual	1.11***	1.11–1.11	0.44***	0.22–0.86	0.99	0.54–1.83	1.31	0.90–1.91
Service manual	0.42***	0.36–0.49	0.29***	0.17–0.47	1.21	0.60–2.43	0.91	0.52–1.58
**Total,** n	6240		6240		15136		15136	

AOR: adjusted odds ratio. Source: KDHS 2022. Significance level:

*** p<0.001.

** p<0.01.

* p<0.05.

® Reference categories.

Assessing tobacco use among women in the multivariable analysis was difficult due to wide confidence intervals, which likely resulted from the low number of women who reported using tobacco in the survey. The results showed that the odds of smoking for women in service-manual occupations are not significantly different from those in agriculture (AOR=1.21, non-significant p-value).

## DISCUSSION

Results from the 2022 KDHS indicate that tobacco control measures in Kenya are beginning to bear fruit since the 2014 KDHS results. For men, the overall smoking and smokeless prevalences decreased from 17.3% and 3.10%, to 12.81% and 2.64%, respectively. This is a 26% drop and is statistically significant, suggesting success in tobacco control measures in the intervening years. While women, still have low smoking and smokeless prevalence at 0.18% and 0.93%, respectively, in 2014, there was a marginal increase in smoking to 0.35%, and smokeless use decreased to 0.77%. There was a reduction in tobacco use across sociodemographic groups including age, marital status and employment in 2022 compared with 2014. While the general decline in prevalence across all age groups aligns with the 2014 KDHS, prevalence is still significantly high for older age groups, with males aged 50–54 years up to twenty times higher in tobacco use compared to males aged 15–19 years in 2022 KDHS, compared to thirteen times higher in 2014 for the same age group comparison. This suggests that older Kenyans are still disproportionately contributing to the prevalence burden than younger individuals, with those aged 30–34 years being the most responsive to reduction efforts. This observation underscores the importance of continued efforts to support cessation among older adults, as well as targeted interventions for younger age groups to further drive down prevalence across the entire population.

The results highlight differences and patterns of tobacco use in Kenya. Smoking is more popular among men than smokeless tobacco use. For both, the odds increase as age increases. The same can be said among women, though smokeless tobacco use is higher than smoking in this group. This is consistent with other findings as to the addictive nature of tobacco that soon after initiation, nicotine addiction makes withdrawal difficult and many find themselves regular smokers, meaning that prevalence increases with age^13,14^. Low prevalence among young adults (<23 years) might suggest that tobacco control measures, such as advertising bans and increased taxes, have been effective, particularly in discouraging initiation among this demographic. Research indicates that tobacco initiation often occurs during adolescence and young adulthood, influenced by factors like advertising and marketing strategies^15-17^. This suggests that these control measures may be successfully countering such influences, contributing to lower prevalence rates in younger individuals.

The results are consistent with a country at the early stages of the tobacco epidemic^18^. High smokeless tobacco use in rural areas with lower incomes is also expected because smokeless tobacco is cheaper, and this accessibility may make it more likely that people will consume it. Also, the likelihood of an individual being poor and uneducated is higher in rural areas and this is likely affecting the divergence in tobacco use patterns between those in urban and rural areas^19,20^.

While the study highlights successful policy interventions, it is important to acknowledge its limitations, particularly in understanding the full scope of tobacco use. The findings point to areas where additional policy efforts are needed, such as in regions where prevalence remains high. For example, even with a decline in prevalence among older Kenyans, this population group still faces a higher risk for tobacco-related diseases and death. Therefore, the support of cessation services for older tobacco users could further accelerate the decline in overall prevalence. These results suggest that existing policies are working and that strengthening them will help ensure a continued decrease in both prevalence and the number of consumers as the population grows.

### Limitations

This study has several limitations that should be considered when interpreting the results. First, the cross-sectional design of the Kenya Demographic and Health Survey (KDHS) data prevents us from establishing a causal relationship between the observed covariates and tobacco use. While the analysis identified strong associations between factors like age, marital status, and education level with tobacco use, it cannot determine whether these factors cause the behavior or if the relationship is influenced by other unmeasured variables.

Second, although our multivariable model included a wide range of socioeconomic and demographic factors, there may be residual confounding from unmeasured variables. The KDHS data, for example, does not collect information on specific tobacco control policies at the county level, the prices of tobacco products, or exposure to tobacco advertising in different regions. The exclusion of these variables from the model means their influence on the prevalence of tobacco use could not be fully accounted for, potentially affecting the precision of our estimates.

## CONCLUSIONS

Results from the 2022 KDHS indicate that tobacco control measures in Kenya are beginning to bear fruit as evidenced by the declines in tobacco use since the 2014 KDHS survey was implemented. There is an apparent reduction in tobacco use across all sociodemographic groups including age, marital status and employment. The overall patterns are mostly consistent with the 2014 KDHS, but the reduction in magnitude is significant.

The effectiveness of tobacco control measures is likely heightened by focusing on the socioeconomic, demographic and geographical factors that affect tobacco control reduction. Given the increased health burden in county budgets as result of increases in NCDs^21^, allocation of resources towards prevention of tobacco use rather than curative treatment of tobacco-induced diseases is critical, including systematic assessment of tobacco use in primary healthcare and offering evidence-based cessation. Therefore, policy makers should strengthen enforcement of existing policies and promote additional policies in compliance with the WHO Framework Convention on Tobacco Control, which provides a wide menu of proven policy interventions. The low prevalence among women is encouraging, but the slight increase in smoking points to the need to focus on prevention with girls and young women to ensure that women in Kenya do not become the next victims of the tobacco epidemic. At the same time, community-based education and campaigns, combined with support for tobacco cessation, will be essential. Finally, there is a need to continuously build the capacity of policy makers at county level on tobacco control. This is because Kenya’s constitution created a two-tier governance structure which share health responsibilities whereby the central/national government is responsible for policy formulation on health issues, while the implementation lies with the county governments.

## Data Availability

The data supporting this research are available from the following source: https://dhsprogram.com/data/
